# Supplementary material for: Prevention of CaCl2-induced aortic inflammation and subsequent aneurysm formation by the CCL3–CCR5 axis
Source: Nat Commun. 2020 Nov 25;11:5994. doi: 10.1038/s41467-020-19763-0 (PMC7688638; doi:10.1038/s41467-020-19763-0)
Supplement: Supplementary file 1 — Supplementary Information [file 41467_2020_19763_MOESM1_ESM.pdf]

# Supplementary Information

# Prevention of $CaCl_2$-induced aortic inflammation and subsequent aneurysm formation by the CCL3-CCR5 axis

Ishida et al.

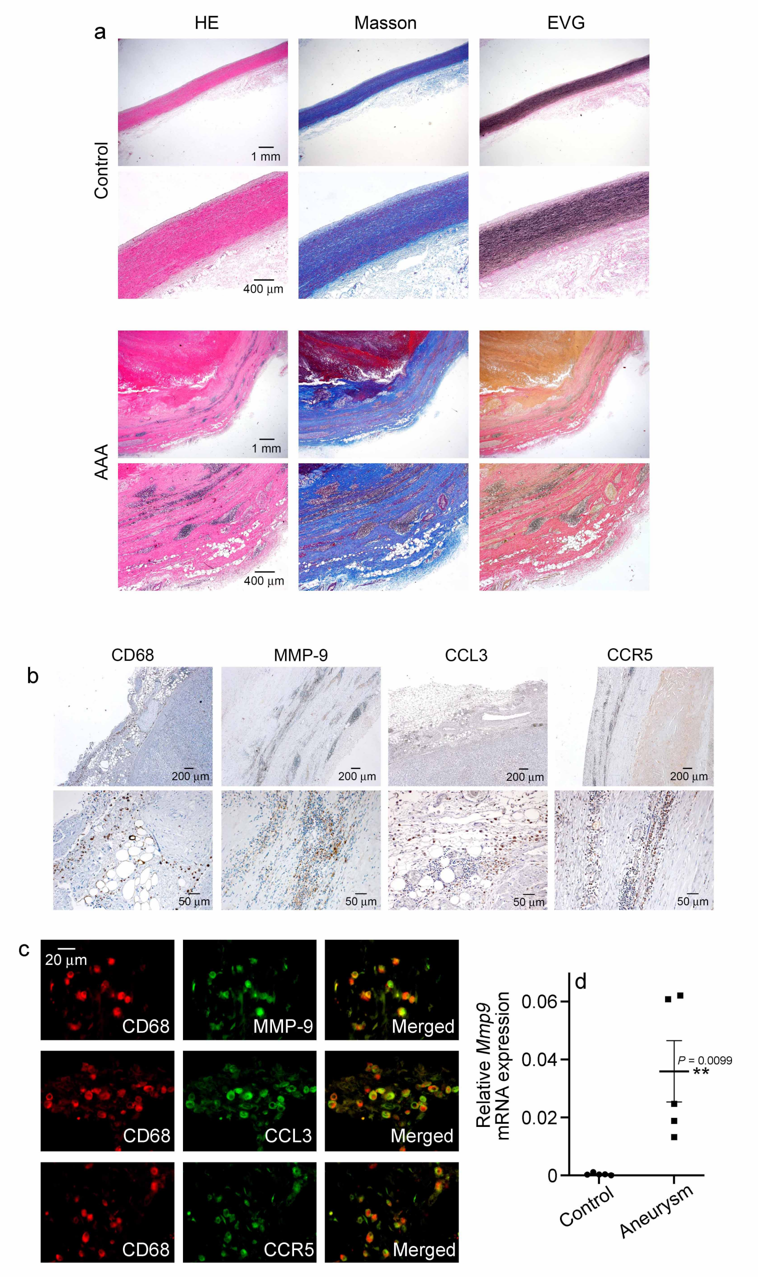


**Supplementary Figure 1.** AAA formation in human. (a) Representative histopathological images of human AAA samples (n=5 independent samples per group). Scale bars, 1 mm (upper panels of Control and AAA), 400 μm (lower panels of Control and AAA). HE, hematoxylin-eosin; MT, Masson trichrome; EVG, Elastica van Gieson. (b) Immunohistochemical analysis of macrophages (CD68), MMP-9, CCL3, and CCR5 proteins in human AAA samples. Scale bars in upper panels, 200 μm. n = 5 independent samples per group. (c) Expression of MMP-9, CCL3, and CCR5 by $CD68^+$ macrophages in the human AAA samples tissues (n= 5 independent samples per group.). Scale bar, 20 μm. (d) Intra-aortic *MMP9* expression of in human AAA samples (n=5 samples in each group). $^{**}P < 0.01$, vs. control. Unpaired two-sided Student's *t* test was used in (d). Data are presented as mean values ± SEM.

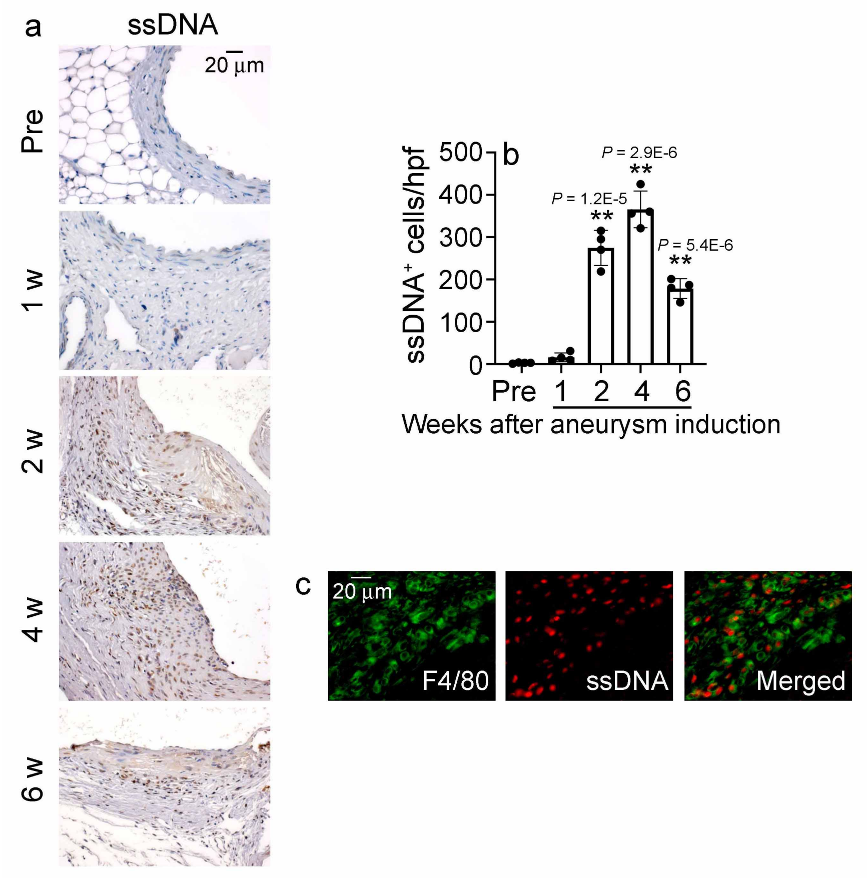


**Supplementary Figure 2.** Detection of apoptotic cells in the aorta of WT mice after $CaCl_2$ treatment (a) Immunohistochemical analysis were performed by using anti-ssDNA antibodies. Representative results from 4 independent experiments are shown (scale bar, 20 µm). (b) Apoptotic cell numbers were determined (n = 4 samples in each time point). ***P*<0.01, vs. pretreatment. (c) A double-color immunofluorescence images using anti-F4/80 and anti-ssDNA. Representative results from 4 independent experiments. Scale bar = 20 µm. One-way ANOVA followed by Dunnett's post-hoc test was used in (b). Data are presented as mean values ± SEM.

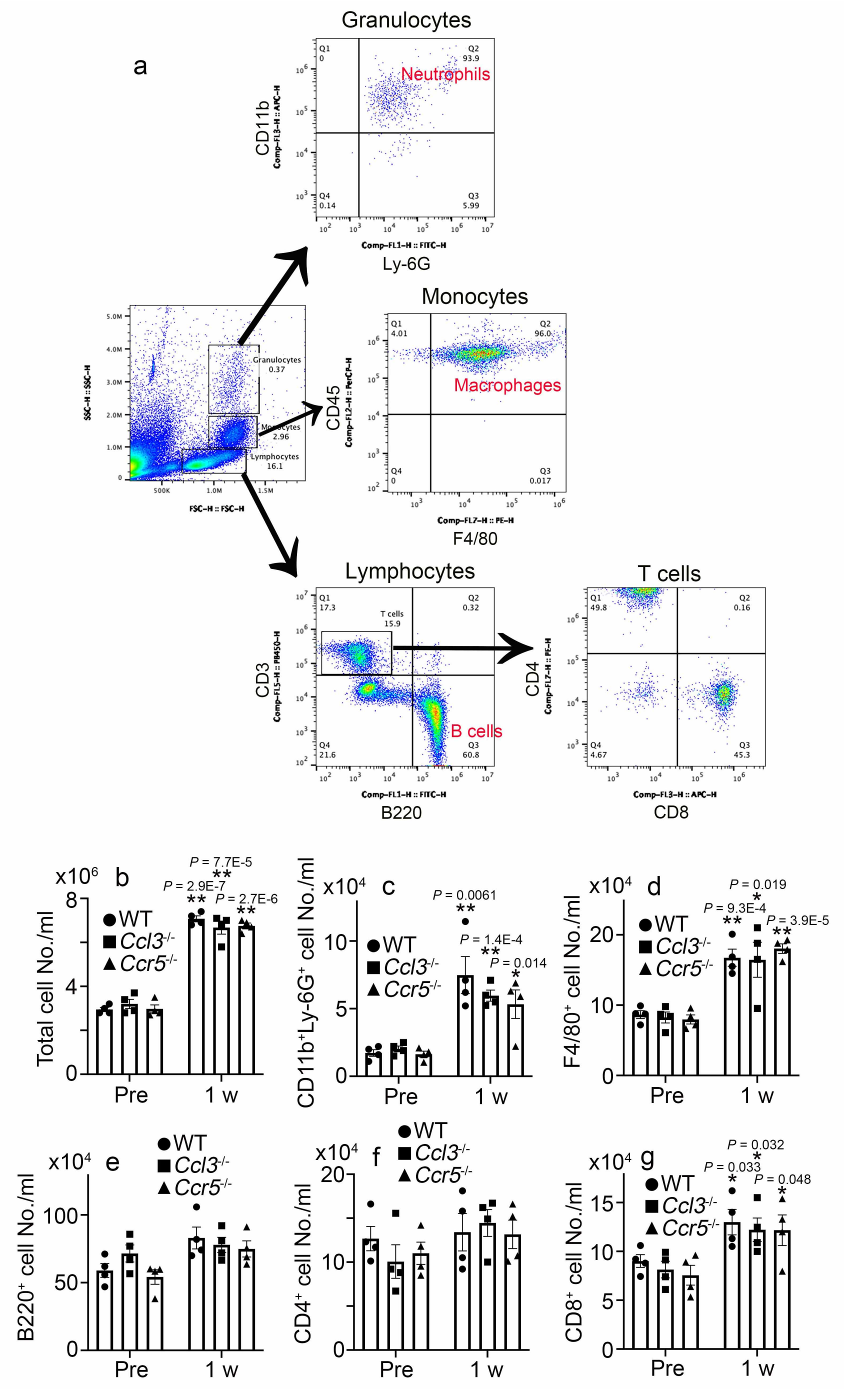


**Supplementary Figure 3.** Circulating immune cells in WT, *Cc3l*$^{-/-}$ and *Ccr5*$^{-/-}$ mice after $CaCl_2$ treatment. (a) Gating strategy to sort neutrophils, macrophages, T cells and B cells. (b to g) The numbers of whole immune cells (b), CD11b$^{+}$Ly6G$^{+}$ cells (c), F4/80$^{+}$ cells (d), B220$^{+}$ cells (e), CD4$^{+}$ cells (f) and CD8$^{+}$ cells (g) were evaluated (n = 4 independent experiments). **$P$<0.01, *$P$<0.05 vs. pretreatment in each strain. Two-way ANOVA followed by Dunnett's post-hoc test was used. Data are presented as mean values ± SEM.

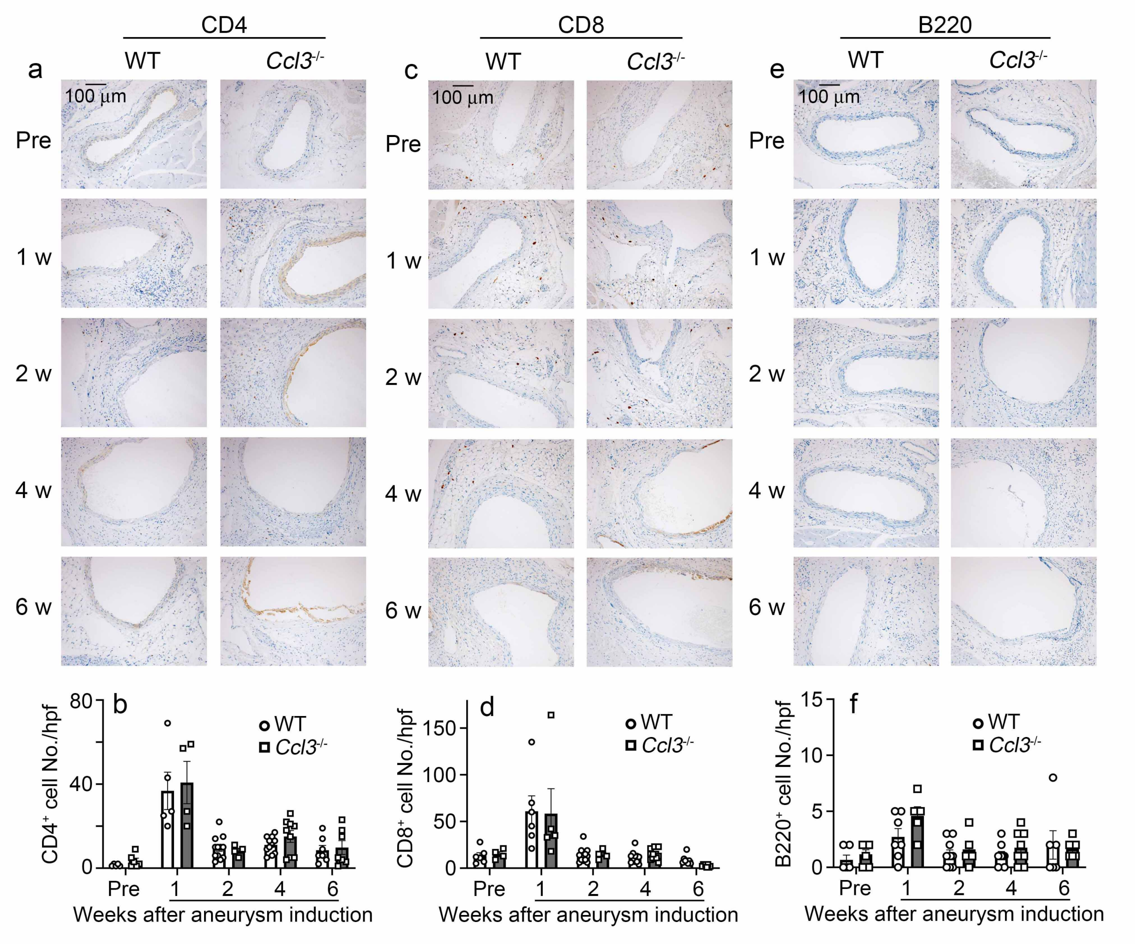


**Supplementary Figure 4.** The accumulation of T cells and B cells into aortic tissues in WT and *Ccl3*$^{-/-}$ mice after $CaCl_2$ treatment. (a, c, e) Representative images of the aorta from WT and *Ccl3*$^{-/-}$ mice. Scale bars, 100 µm. (b, d, f) The numbers of CD4$^+$, CD4$^+$ and B220$^+$ cells were measured. (b) CD4$^+$ (Pre: n = 5 in WT, n = 6 in *Ccl3*$^{-/-}$; 1 w: n = 5 in WT, n = 4 in *Ccl3*$^{-/-}$; 2 w: n = 12 in WT, n = 4 in *Ccl3*$^{-/-}$; 4 w: n = 11 in WT, n = 9 in *Ccl3*$^{-/-}$; 6 w: n = 7 each in WT and *Ccl3*$^{-/-}$). (d) CD8$^+$ T cells (Pre: n = 6 in WT, n = 4 in *Ccl3*$^{-/-}$; 1 w: n = 6 in WT, n = 5 in *Ccl3*$^{-/-}$; 2 w: n = 12 in WT, n = 4 in *Ccl3*$^{-/-}$; 4 w: n = 11 in WT, n = 12 in *Ccl3*$^{-/-}$; 6 w: n = 8 each in WT and *Ccl3*$^{-/-}$). (f) B220$^+$ B cells (Pre: n = 6 each in WT and *Ccl3*$^{-/-}$; 1 w: n = 7 in WT, n = 5 in *Ccl3*$^{-/-}$; 2 w: n = 10 in WT, n = 5 in *Ccl3*$^{-/-}$; 4 w: n = 9 in WT, n = 8 in *Ccl3*$^{-/-}$; 6 w: n = 6 each in WT and *Ccl3*$^{-/-}$). Data are presented as mean values ± SEM.

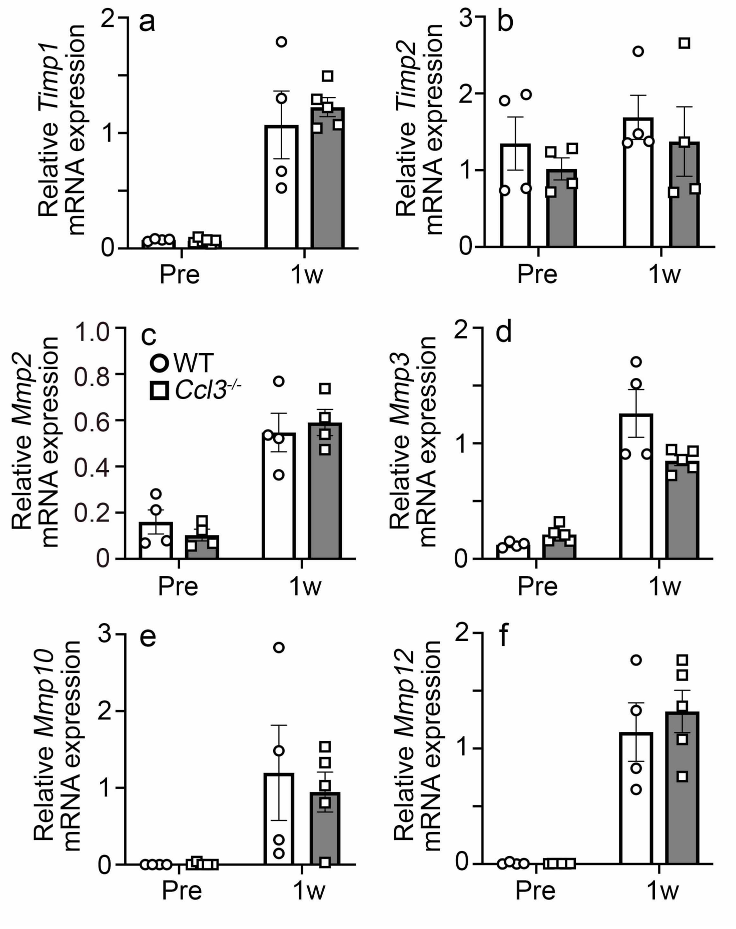


**Supplementary Figure 5.** Gene expression of *Timps* and *Mmps* in aortic tissues from WT and *Ccl3*$^{-/-}$ mice after $CaCl_2$ treatment. Intra-aortic gene expression of (a)*Timp1* (Pre: n = 4 in WT, n = 6 in *Ccl3*$^{-/-}$; 1 w: n = 4 in WT, n = 5 in *Ccl3*$^{-/-}$), (b) *Timp2* (Pre: n = 4 each in WT and *Ccl3*$^{-/-}$; 1 w: n = 4 each in WT and *Ccl3*$^{-/-}$), (c) *Mmp2* (Pre: n = 4 each in WT and *Ccl3*$^{-/-}$; 1 w: n = 4 each in WT and *Ccl3*$^{-/-}$), (d) *Mmp3* (Pre: n = 4 in WT, n = 6 in *Ccl3*$^{-/-}$; 1 w: n = 4 in WT, n = 5 in *Ccl3*$^{-/-}$), (e) *Mmp10* (Pre: n = 4 in WT, n = 5 in *Ccl3*$^{-/-}$; 1 w: n = 4 in WT, n = 5 in *Ccl3*$^{-/-}$) and (f) *Mmp12* (Pre: n = 4 in WT, n = 6 in *Ccl3*$^{-/-}$; 1 w: n = 4 in WT, n = 5 in *Ccl3*$^{-/-}$). Data are presented as mean values ± SEM.

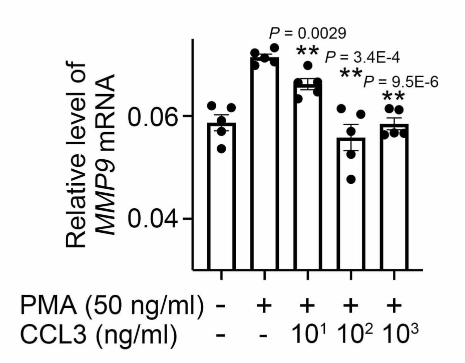


**Supplementary Figure 6.** Regulation of MMP-9 expression in THP-1 cells (a human macrophage cell line) by CCL3. THP-1 cells were stimulated with PMA for 24 h to be subjected to qRT-PCR. The effects of CCL3 on the gene expression of *Mmp9* in PMA-stimulated THP cells were examined by qRT-PCR (n = 4 independent experiments). $^{**}P < 0.01$, $^{*}P < 0.05$, vs. PMA only. One-way ANOVA followed by Dunnett's post hoc test was used. Data are presented as mean values ± SEM.

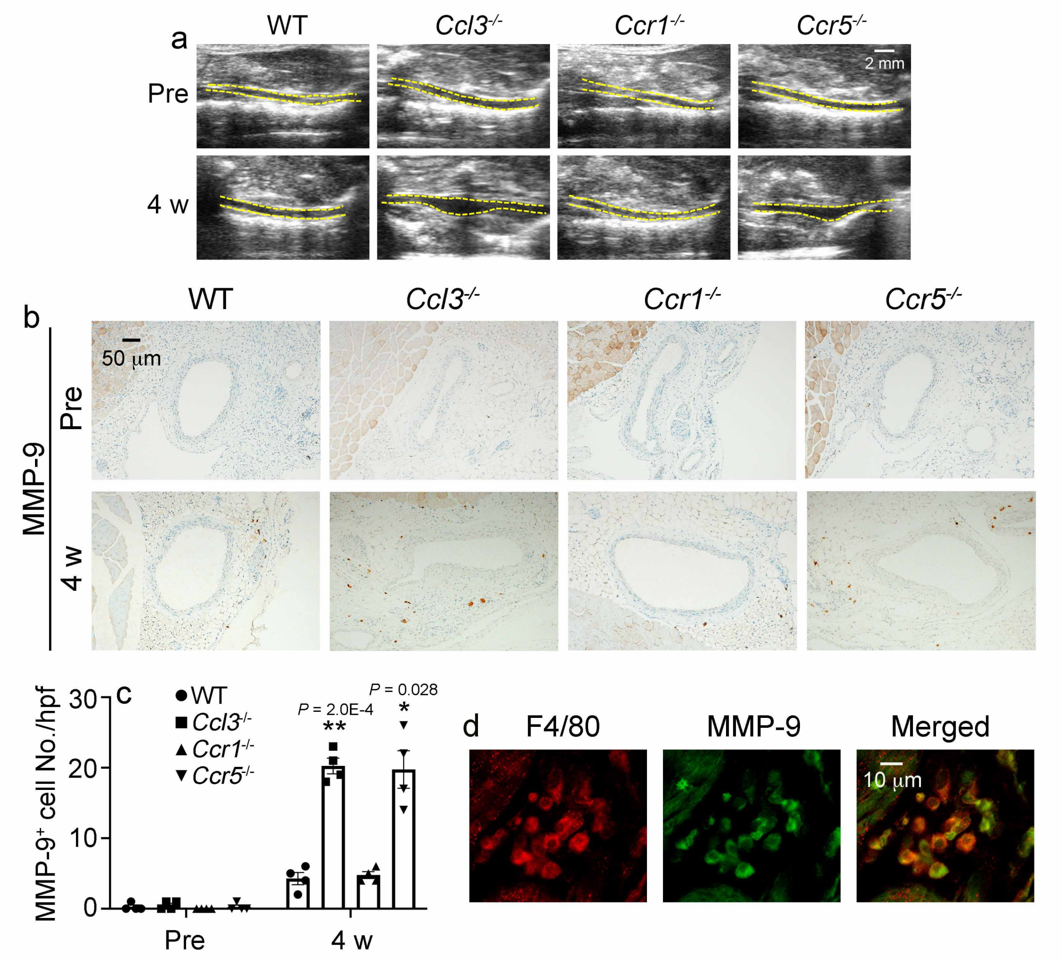


**Supplementary Figure 7.** The roles of the CCL3-CCR5 axis on Ang II-induced AAA formation. (a) Representative transabdominal ultrasound images showing increases in the luminal expansion of abdominal aorta in *Ccl3*$^{-/-}$ and *Ccr5*$^{-/-}$ mice (6 independent experiments). Dashed yellow lines outline the lumen. (b) Immunohistochemical images of MMP-9 expression in aorta samples (6 independent experiments). Scale bar, 50 μm. (c) The number of MMP-9$^{+}$ cells were determined (n = 4 in each mouse strain). (d) Representative double-color immunofluorescence images of MMP-9 expression by F4/80$^{+}$ macrophages in aortic tissues of WT mice at 4 weeks after Ang II infusion (4 independent experiments). Scale bar, 10 μm. Unpaired two-sided Student's *t* test was used in (c).Data are presented as mean values ± SEM.

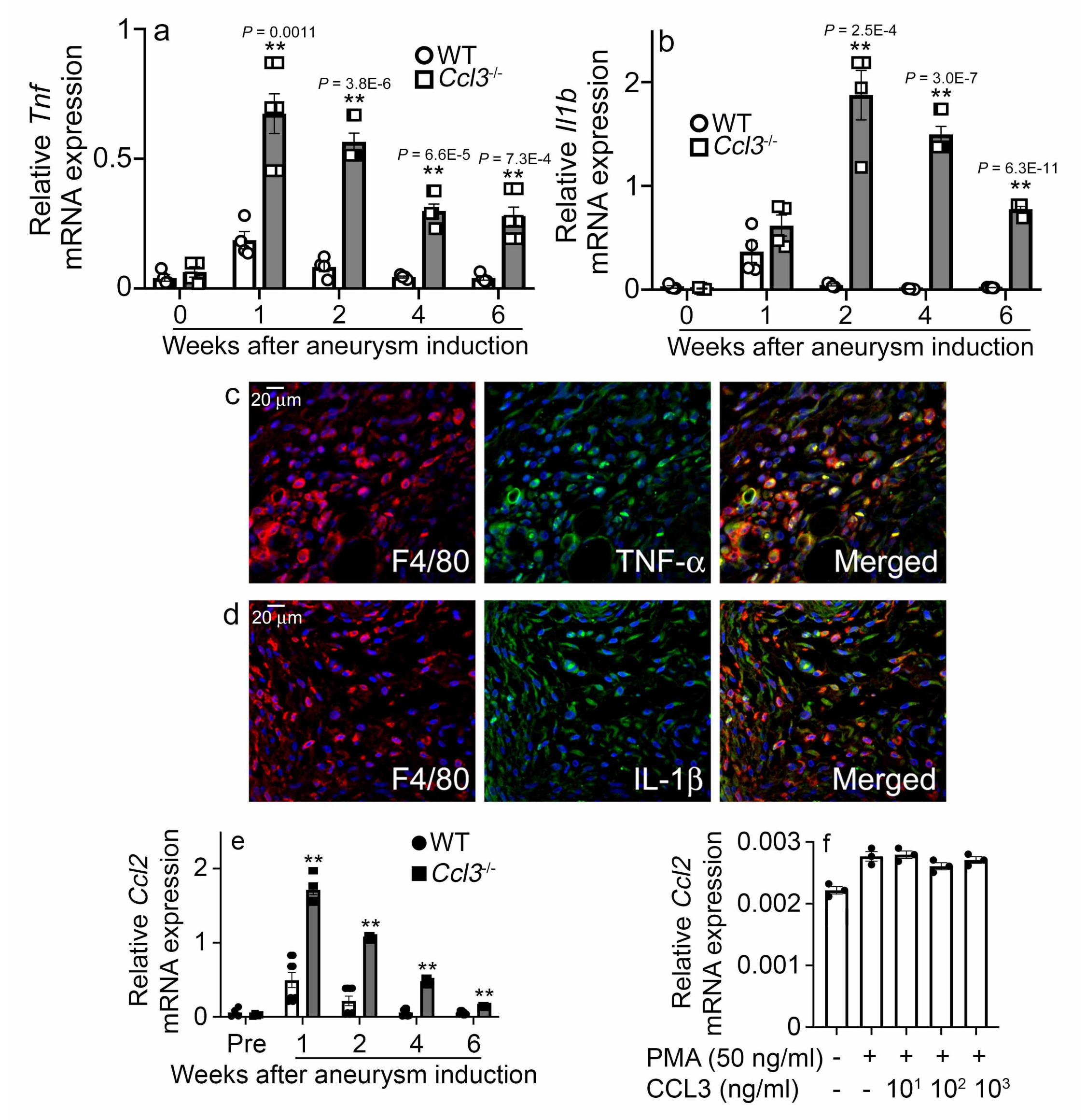


**Supplementary Figure 8.** Expression of TNF-α and IL-1β in mouse aorta. (a and b) Intra-aortic gene expression of (a) *Tnf* (Pre: n = 4 each in WT and *Ccl3*$^{-/-}$;1, 2, 4 and 6 w: n = 4 in WT, n = 6 in *Ccl3*$^{-/-}$) and (b) *Il1b* (Pre, 1 and 2 w: n = 4 each in WT and *Ccl3*$^{-/-}$; 4 w: n = 4 in WT, n = 6 in *Ccl3*$^{-/-}$; 6 w: 6 each in , in WT and *Ccl3*$^{-/-}$). $^{**}P < 0.01$, vs. WT mice, by 2-way ANOVA followed by Dunnett's post-hoc test. (c and d) Double-color immunofluorescence images of (c) TNF-α- or (d) IL-1β-expressing cells in the AAA tissues (representative results from 4 independent experiments). Scale bar = 20 μm. Blue, nuclear staining by DAPI. (e) Intra-aortic *Ccl2* expression in WT and *Ccl3*$^{-/-}$ mice after $CaCl_2$ treatment (Pre: n = 4 each in WT and *Ccl3*$^{-/-}$;1 w: n = 8 each in WT and *Ccl3*$^{-/-}$; and, 2, 4 and 6 w: n = 8 in WT, n = 6 in *Ccl3*$^{-/-}$). $^{**}P < 0.01$, vs. WT mice. (f) The effects of CCL3 on *Ccl2* expression of in PMA-stimulated WT macrophages (n = 3 independent experiments). Two-way ANOVA followed by Dunnett's post-hoc test was used in (a), (b) and (e). Data are presented as mean values ± SEM.

**Supplementary Table 1**. Change of aortic diameter in WT and *Ccl3*$^{-/-}$ mice after $CaCl_2$ treatment.

| | WT | *Ccl3*$^{-/-}$ |
|---|---|---|
| Pretreatment (μm) | 540 ± 4.7 (n = 9) | 556 ± 16.1 (n = 8) |
| Posttreatment (μm) | 725 ± 15.4$^{A}$ (n = 10) | 980 ± 89.4$^{A,B}$ (n = 8) |
| AAA development | 4/10 (40%) | 7/8 (87%) |
| % increase | 34 ± 2.9 | 76 ± 16.1$^{C}$ |

**Supplementary Table 1. Change of aortic diameter in WT and *Ccl3*$^{-/-}$ mice after $CaCl_2$ treatment.** The development of AAA was defined as more than 35% increase relative to the mean aortic diameter of pretreatment mice in each group. The percent increase is represented as a percent change compared with the mean value of pretreatment. $^{A}P < 0.01$, vs. pretreatment; $^{B}P < 0.01$, vs. $CaCl_2$-treated WT; $^{C}P < 0.05$, vs. $CaCl_2$-treated WT, by unpaired two-sided Student's *t* test. Data are presented as mean values ± SEM.

**Supplementary Table 2**. Change of aortic diameter in $CaCl_2$-treated WT mice after anti-CCL3 antibody administration.

| | control IgG | anti-CCL3 |
|---|---|---|
| Pretreatment (μm) | 530 ± 5.7 (n = 5) | 511 ± 4.4 (n = 5) |
| Posttreatment (μm) | 648 ± 20.1$^{A}$ (n = 4) | 723 ± 18.5$^{A,B}$ (n = 8) |
| AAA development | 0/4 (0%) | 6/8 (75%) |
| % increase | 22 ± 3.8 | 41± 3.6$^{C}$ |

**Supplementary Table 2. Change of aortic diameter in $CaCl_2$-treated WT mice after anti-CCL3 antibody administration.**

The development of AAA was defined as more than 35% increase relative to the mean aortic diameter of pretreatment mice in each group. The percent increase is represented as a percent change compared with the mean value of pretreatment. $^{A}P < 0.01$, vs. pretreatment; $^{B}P < 0.05$, vs. $CaCl_2$-treated mice with control IgG; $^{C}P < 0.01$, vs. $CaCl_2$-treated mice with control IgG, by unpaired two-sided Student's *t* test. Data are presented as mean values ± SEM.

**Supplementary Table 3**. Change of aortic diameter in BM-chimeric mice after $CaCl_2$ treatment.

| | WT-BM in WT | *Ccl3*$^{-/-}$-BM in WT | WT-BM in *Ccl3*$^{-/-}$ | *Ccl3*$^{-/-}$-BM in *Ccl3*$^{-/-}$ |
|---|---|---|---|---|
| Pretreatment (μm) | 543 ± 3.1 (n = 6) | 539 ± 7.2 (n = 6) | 543 ± 12.2 (n = 6) | 551 ± 8.7 (n = 6) |
| Posttreatment (μm) | 700 ± 11.9$^{A}$ (n = 6) | 848 ± 64.4$^{A,B}$ (n = 6) | 693 ± 20.3$^{A}$ (n = 6) | 875 ± 26.7$^{A,B}$ (n = 6) |
| AAA development | 0/6 (0%) | 6/6 (100%) | 1/6 (16%) | 6/6 (100%) |
| % increase | 29 ± 2.2 | 57 ± 12.0$^{C}$ | 28 ± 3.7 | 59 ± 4.9$^{C}$ |

**Supplementary Table 3. Change of aortic diameter in BM-chimeric mice after $CaCl_2$ treatment.** The development of AAA was defined as more than 35% increase relative to the mean aortic diameter of pretreatment mice in each group. The percent increase is represented as a percent change compared with the mean value of pretreatment. $^{A}P < 0.05$, vs. pretreatment; $^{B}P < 0.05$, vs. WT-BM in WT after $CaCl_2$ treatment; $^{C}P < 0.05$, vs. WT-BM in WT after $CaCl_2$ treatment, by one-way ANOVA followed by Dunnett's post-hoc test. Data are presented as mean values ± SEM.

**Supplementary Table 4**. Change of aortic diameter in WT, *Ccr1*$^{-/-}$ and *Ccr5*$^{-/-}$ mice after $CaCl_2$ treatment.

| | WT | *Ccr1*$^{-/-}$ | *Ccr5*$^{-/-}$ |
|---|---|---|---|
| Pretreatment (μm) | 547 ± 9.7 (n = 6) | 567 ± 9.6 (n = 6) | 566 ± 26.2 (n = 6) |
| Posttreatment (μm) | 711 ± 16.0$^{A}$ (n = 7) | 644 ± 10.6$^{A}$ (n = 7) | 876 ± 41.2$^{A,B}$ (n = 8) |
| AAA development | 2/7 (78%) | 0/7 (0%) | 8/8 (100%) |
| % increase | 30 ± 2.9 | 14 ± 1.9 | 55 ± 7.3$^{C}$ |

**Supplementary Table 4. Change of aortic diameter in WT, *Ccr1*$^{-/-}$ and *Ccr5*$^{-/-}$ mice after $CaCl_2$ treatment.**
The development of AAA was defined as more than 35% increase relative to the mean aortic diameter of pretreatment mice in each group. The percent increase is represented as a percent change compared with the mean value of pretreatment. $^{A}P < 0.01$, vs. pretreatment; $^{B}P < 0.05$, vs. $CaCl_2$-treated WT; $^{C}P < 0.01$, vs. $CaCl_2$-treated WT, by one-way ANOVA followed by Dunnett's post-hoc test. Data are presented as mean values ± SEM.

**Supplementary Table 5**. Change of aortic diameter in $CaCl_2$-treated mice after CCL3 administration.

| | WT | | *Ccl3*$^{-/-}$ | | *Ccr5*$^{-/-}$ | |
|---|---|---|---|---|---|---|
| | PBS | CCL3 | PBS | CCL3 | PBS | CCL3 |
| Pretreatment (μm) | 547 ± 5.1 (n = 6) | 524 ± 5.0 (n = 6) | 544 ± 2.6 (n = 6) | 551 ± 6.1 (n = 6) | 528 ± 7.5 (n = 6) | 541 ± 2.1 (n = 6) |
| Posttreatment (μm) | 755 ± 25.0$^{A}$ (n = 6) | 563 ± 12.3$^{A,B}$ (n = 6) | 933 ± 48.2$^{A}$ (n = 6) | 583± 28.5$^{B}$ (n = 6) | 878 ± 52.3$^{A}$ (n = 6) | 871 ± 27.0$^{A}$ (n = 6) |
| AAA development | 3/6 (50%) | 0/6 (0%) | 6/6 (100%) | 0/6 (0%) | 6/6 (100%) | 6/6 (100%) |
| % increase | 38 ± 4.6 | 7.3± 2.3$^{C}$ | 72 ± 8.9 | 5.7 ± 5.2$^{C}$ | 66 ± 9.9 | 61 ± 5.0 |

**Supplementary Table 5. Change of aortic diameter in $CaCl_2$-treated mice after CCL3 administration.**
The development of AAA was defined as more than 35% increase relative to the mean aortic diameter of pretreatment mice in each group. The percent increase is represented as a percent change compared with the mean value of pretreatment. $^{A}P < 0.05$, vs. pretreatment in each group; $^{B}P < 0.01$, vs. PBS treatment in each strain; $^{C}P < 0.05$, vs. PBS treatment in each strain, by unpaired two-sided Student's *t* test. Data are presented as mean values ± SEM.

**Supplementary Table 6.** Sequences of the mouse primers used for real-time RT-PCR.

| Transcript | Sequence |
|---|---|
| *Ccl3* | (F) 5’-TGAAACCAGCAGCCTTTGCTC-3’<br>(R) 5’-AGGCATTCAGTTCCAGGTCAGTG-3’ |
| *Ccl2* | (F) 5’-GCATCCACGTGTTGGCTCA-3’<br>(R) 5’-CTCCAGCCTACTCATTGGGATCA-3’ |
| *Il1b* | (F) 5’-TCCAGGATGAGGACATGAGCAC-3’<br>(R) 5’-GAACGTCACACACCAGCAGGTTA-3’ |
| *Tnf* | (F) 5’-AAGCCTGTAGCCCACGTCGTA-3’<br>(R) 5’-GGCACCACTAGTTGGTTGTCTTTG-3’ |
| *Timp1* | (F) 5’-GTCCCAGAACCGCAGTGAAGA-3’<br>(R) 5’-CTGCAGGCACTGATGTGCAA-3’ |
| *Timp2* | (F) 5’-TCCTGTGCGGTGGTATCAATATGTA-3’<br>(R) 5’-GCTCACGGCGAACATCAGAA-3’ |
| *Mmp2* | (F) 5’- GATAACCTGGATGCCGTCGTG-3’<br>(R) 5’- CTTCACGCTCTTGAGACTTTGGTTC-3’ |
| *Mmp3* | (F) 5’-CTGGACCAGGGATTAATGGAGA -3’<br>(R) 5’- TCATGAGCAGCAACCAGGAA-3’ |
| *Mmp9* | (F) 5’- GCCCTGGAACTCACACGACA-3’<br>(R) 5’- TTGGAAACTCACACGCCAGAAG-3’ |
| *Mmp10* | (F) 5’- ATTGGAATCCCGAGCCTGAA-3’<br>(R) 5’- CTCGGACTGCCCAGAACTGA-3’ |
| *Mmp12* | (F) 5’-GATGGATGAAGCGGTACCTCACTTA-3’<br>(R) 5’-GAGTCACATCACTCCAGACTTGGAA-3’ |
| *Nos2* | (F) 5’-GCAGAGATTGGAGGCCTTGTG-3’<br>(R) 5’-GGGTTGTTGCTGAACTTCCAGTC-3’ |
| *Cd206* | (F) 5’- AGCTTCATCTTCGGGCCTTTG-3’<br>(R) 5’- GGTGACCACTCCTGCTGCTTTAG-3’ |
| *Actb* | (F) 5’- CATCCGTAAAGACCTCTATGCCAAC-3’<br>(R) 5’- ATGGAGCCACCGATCCACA-3’ |

**Supplementary Table 6. Sequences of the mouse primers used for real-time RT-PCR.**

(F), Forward primer; (R), Reverse primer

**Supplementary Table 7.** Sequences of the human primers used for real-time RT-PCR.

| Transcript | Sequence |
|---|---|
| *Mmp9* | (F) 5'-ACGCACGACGTCTTCCAGTA-3'<br>(R) 5'-CCACCTGGTTCAACTCACTCC-3' |
| *Actb* | (F) 5'-TGGCACCCAGCACAATGAA-3'<br>(R) 5'-CTAAGTCATAGTCCGCCTAGAAGCA-3' |

**Supplementary Table 7. Sequences of the human primers used for real-time RT-PCR.**

(F), Forward primer; (R), Reverse primer
