# Supplementary material for: Prevention of CaCl2-induced aortic inflammation and subsequent aneurysm formation by the CCL3–CCR5 axis
Source: Nat Commun. 2020 Nov 25;11:5994. doi: 10.1038/s41467-020-19763-0 (PMC7688638; doi:10.1038/s41467-020-19763-0)
Supplement: Supplementary file 3 — Reporting Summary [file 41467_2020_19763_MOESM3_ESM.pdf]

nature research

Corresponding author(s): Toshikazu Kondo

Last updated by author(s): Oct 21, 2020

Reporting Summary

Nature Research wishes to improve the reproducibility of the work that we publish. This form provides structure for consistency and transparency in reporting. For further information on Nature Research policies, see our Editorial Policies and the Editorial Policy Checklist.

Statistics

For all statistical analyses, confirm that the following items are present in the figure legend, table legend, main text, or Methods section.

n/a	Confirmed	
☐	☒	The exact sample size (*n*) for each experimental group/condition, given as a discrete number and unit of measurement
☐	☒	A statement on whether measurements were taken from distinct samples or whether the same sample was measured repeatedly
☐	☒	The statistical test(s) used AND whether they are one- or two-sided<br>*Only common tests should be described solely by name; describe more complex techniques in the Methods section.*
☐	☒	A description of all covariates tested
☐	☒	A description of any assumptions or corrections, such as tests of normality and adjustment for multiple comparisons
☐	☒	A full description of the statistical parameters including central tendency (e.g. means) or other basic estimates (e.g. regression coefficient) AND variation (e.g. standard deviation) or associated estimates of uncertainty (e.g. confidence intervals)
☐	☒	For null hypothesis testing, the test statistic (e.g. *F*, *t*, *r*) with confidence intervals, effect sizes, degrees of freedom and *P* value noted<br>*Give P values as exact values whenever suitable.*
☒	☐	For Bayesian analysis, information on the choice of priors and Markov chain Monte Carlo settings
☒	☐	For hierarchical and complex designs, identification of the appropriate level for tests and full reporting of outcomes
☒	☐	Estimates of effect sizes (e.g. Cohen's *d*, Pearson's *r*), indicating how they were calculated

Our web collection on statistics for biologists contains articles on many of the points above.

Software and code

Policy information about availability of computer code

Data collection	Excel
Data analysis	Statcel3, FlowJo

For manuscripts utilizing custom algorithms or software that are central to the research but not yet described in published literature, software must be made available to editors and reviewers. We strongly encourage code deposition in a community repository (e.g. GitHub). See the Nature Research guidelines for submitting code & software for further information.

Data

Policy information about availability of data

All manuscripts must include a data availability statement. This statement should provide the following information, where applicable:

- Accession codes, unique identifiers, or web links for publicly available datasets
- A list of figures that have associated raw data
- A description of any restrictions on data availability

The source data underlying Figs. 1–8 are provided as a Source Data file. All other data supporting the findings of this study are available within the article and its Supplementary Information and from the corresponding author upon reasonable request. A reporting summary for this article is available as a Supplementary Information file.

Field-specific reporting

Please select the one below that is the best fit for your research. If you are not sure, read the appropriate sections before making your selection.

- [x] Life sciences
- [ ] Behavioural & social sciences
- [ ] Ecological, evolutionary & environmental sciences

For a reference copy of the document with all sections, see nature.com/documents/nr-reporting-summary-flat.pdf

Life sciences study design

All studies must disclose on these points even when the disclosure is negative.

Sample size	Statistical methods were used to predetermined sample size in vivo and in vitro analyses. We designed in vivo experiments to ensure that minimum number of mice are used to obtain biologically significant results.
Data exclusions	No data was excluded from the analysis.
Replication	The number of independent experiments is specified in each figure legend, with at least 3 independent experiments, unless otherwise specified.
Randomization	Animals with similar age and weight were randomly allocated to experimental groups. For in vitro experiments, cultures were randomly chosen for different treatments and experiments were performed multiple times.
Blinding	Investigators were blinded to group allocation during data collection and/or analysis.

Reporting for specific materials, systems and methods

We require information from authors about some types of materials, experimental systems and methods used in many studies. Here, indicate whether each material, system or method listed is relevant to your study. If you are not sure if a list item applies to your research, read the appropriate section before selecting a response.

Materials & experimental systems

n/a	Involved in the study	
☐	☒	Antibodies
☐	☒	Eukaryotic cell lines
☒	☐	Palaeontology and archaeology
☐	☒	Animals and other organisms
☐	☒	Human research participants
☒	☐	Clinical data
☒	☐	Dual use research of concern

Methods

n/a	Involved in the study	
☒	☐	ChIP-seq
☐	☒	Flow cytometry
☒	☐	MRI-based neuroimaging

Antibodies

Antibodies used

The following monoclonal antibodies (mAbs) and polyclonal antibodies (pAbs) were used for immunohistochemical and immunofluorescence analysis: rabbit anti-human CCL3 pAbs (GTX52609, Gene Tex, Irvine, CA, 1:50 dilution), mouse anti-human CD68 mAb (clone PG-M1, M0876, DAKO, Glostrup, Denmark, 1:100 dilution), goat anti-human MMP-9 pAbs (sc-6840, Santa Cruz, Dallas, TX, 1:50 dilution), rabbit anti-human CCL3/MIP-1alpha pAbs (GTX52609, Gene Tex, Irvine, CA, 1:100 dilution), goat anti-human CCR5 pAbs (GTX21673, Gene Tex, Irvine, CA, 1:300 dilution), goat anti-mouse CCL3 pAbs (GTX10381, Gene Tex, Irvine, CA, 1:80 dilution), goat anti-mouse CCR5 pAbs (sc-6129, 1:50 dilution), goat anti-mouse MMP-9 pAbs (sc-6840, 1:100 dilution) (Santa Cruz Biotechnology, Santa Cruz, CA), rat anti-mouse F4/80 mAb (clone BM8, T-2028, BMA Biomedicals, Switzerland, 1:50 dilution), rat anti-human CD3 mAb which cross-reacts with mouse CD3 (clone CD3-12, MCA1477, AbD Serotec, Raleigh, NC, 1:70 diluted), rat anti-mouse B220/CD45R mAb (clone RM0063-9F14, NBP2-12168, Novs Biologicals, Centennial, CO, 1:100 diluted), rabbit anti-mouse CD4 mAb (clone EPR19514, ab183685, Abcam, Cambridge, UK, 1:200 diluted), rat anti-mouse CD8 pAb (ab203035, Abcam, Cambridge, UK, 1:100 diluted), rabbit anti-mouse NOS2 pAbs (sc-650, Santa Cruz, Dallas, TX, 1:200 diluted), rabbit anti-mouse CD206 pAbs (ab64693, Abcam, Cambridge, UK, 1:1000 diluted), goat anti-mouse TNF-a pAbs (sc-1350, 1:100 diluted), rabbit anti-mouse IL-1[illegible] pAbs (sc-7884, 1:200 diluted) (SantaCruz, Dallas, TX), anti-ssDNA (IBL, Japan, 1:50 diluted), Cy3-conjugated donkey anti-rat IgG pAbs (712-165-153, 1:200 diluted), -goat IgG pAbs (705-165-147, 1:100 diluted), and -rabbit IgG pAbs (711-165-152, 1:200 diluted), and FITC-conjugated donkey anti-goat IgG pAbs (705-095-147, 1:50 diluted), -rabbit IgG pAbs (711-095-152, 1:50 diluted), and -rat IgG pAbs (712-095-153, 1:50 diluted) (Jackson Immunoresearch Laboratories, West Grove, PA).

The following antibodies were used for Western blotting analysis: rabbit anti-ERK mAb (1:1000, #4695), rabbit anti-phosphorylated (p)-ERK mAb (1:2000, #4370), rabbit anti-p38 pAbs (1:1000, #9212), rabbit anti-p-p38 mAb (1:1000, #4511), rabbit anti-JNK pAbs (1:1000, #9252), rabbit anti-p-JNK pAbs (1:1000, #9251), and rabbit anti-GAPDH mAb (1:1000, #5174) obtained from Cell Signaling, Danvers, MA.

The following antibodies were used for flow cytometry: CD11b (1:100, clone M1/70, 20-0112, Tonbo Biosciences, San Diego, CA), Ly-6G (1:100, clone RB6-8C5, 35-5931, Tonbo Biosciences), F4/80 (1:80, clone BM8.1, 50-4801, Tonbo Biosciences), B220 (1:50, clone RA3-6B2, 103205, BioLegend, San Diego, CA), CD3 (1:40, clone 17A2, 75-0032, Tonbo Biosciences), CD4 (1:80, RM4-5, 50-0042, Tonbo Biosciences), CD8 (1:80, clone 53-6.7, 20-0081, Tonbo Biosciences), and CD45 (1:80, clone 30-F11, 103131, BioLegend).

Validation

The antibodies are from commercial sources. For validation, the following methods were used: 1) use of isotype controls for analyses, 2) results from previous publications from our lab, 3) manufacture provided validation on the same species, relevant information on the antibodies are available on the manufacturers' websites.
rabbit anti-human CCL3 pAbs (GTX52609, Gene Tex, Irvine, CA), https://www.genetex.com/Product/Detail/MIP1-alpha-antibody/GTX52609
mouse anti-human CD68 mAb (clone PG-M1, M0876, DAKO, Glostrup, Denmark), https://www.agilent.com/en/product/immunohistochemistry/antibodies-controls/primary-antibodies/cd68-(concentrate)-76550
goat anti-human MMP-9 pAbs (sc-6840, Santa Cruz, Dallas, TX), https://www.scbt.com/p/mmp-9-antibody-c-20?productCanUrl=mmp-9-antibody-c-20&_requestid=739477
rabbit anti-human CCL3/MIP-1alpha pAbs (GTX52609, Gene Tex, Irvine, CA), https://www.genetex.com/Product/Detail/MIP1-alpha-antibody/GTX52609
goat anti-human CCR5 pAbs (GTX21673, Gene Tex, Irvine, CA), https://www.genetex.com/Product/Detail/CCR5-antibody/GTX21673
goat anti-mouse CCL3 pAbs (GTX10381, Gene Tex, Irvine, CA), https://www.genetex.com/Product/Detail/MIP1-alpha-antibody/GTX10381
goat anti-mouse CCR5 pAbs (sc-6129), https://www.citeab.com/antibodies/781428-sc-6129-ckr-5-antibody-m-20
goat anti-mouse MMP-9 pAbs (sc-6840) (Santa Cruz Biotechnology, Santa Cruz, CA), https://www.scbt.com/p/mmp-9-antibody-c-20?productCanUrl=mmp-9-antibody-c-20&_requestid=739672
rat anti-mouse F4/80 mAb (clone BM8, T-2028, BMA Biomedicals, Switzerland), http://www.bma.ch/en/products/t-2028
rat anti-human CD3 mAb which cross-reacts with mouse CD3 (clone CD3-12, MCA1477, AbD Serotec, Raleigh, NC), https://www.biocompare.com/9776-Antibodies/114007-RAT-ANTI-HUMAN-CD3/
rat anti-mouse B220/CD45R mAb (clone RM0063-9F14, NBP2-12168, Novs Biologicals, Centennial, CO), https://www.biocompare.com/9776-Antibodies/3054146-CD45R-Antibody/?pda=9776%7C3054146_39_0%7C%7C%7CB220
rabbit anti-mouse CD4 mAb (clone EPR19514, ab183685, Abcam, Cambridge, UK), https://www.citeab.com/antibodies/3342807-ab183685-anti-cd4-antibody-epr19514
rat anti-mouse CD8 pAb (ab203035, Abcam, Cambridge, UK), https://www.citeab.com/antibodies/4637276-ab203035-anti-cd8-antibody
rabbit anti-mouse NOS2 pAbs (sc-650, Santa Cruz, Dallas, TX), https://www.scbt.com/p/nos2-antibody-m-19?productCanUrl=nos2-antibody-m-19&_requestid=740342
rabbit anti-mouse CD206 pAbs (ab64693, Abcam, Cambridge, UK), https://www.citeab.com/antibodies/737348-ab64693-anti-mannose-receptor-antibody
goat anti-mouse TNF-a pAbs (sc-1350), rabbit anti-mouse IL-1b pAbs (sc-7884) (SantaCruz, Dallas, TX), https://www.citeab.com/antibodies/800587-sc-7884-il-1-antibody-h-153
anti-ssDNA (IBL, Japan), https://www.ibl-international.com/en/single-stranded-dna-ssdna-antibody
Cy3-conjugated donkey anti-rat IgG pAbs (712-165-153), https://www.jacksonimmuno.com/catalog/products/712-165-153
Cy3-conjugated donkey anti-goat IgG pAbs (705-165-147), https://www.jacksonimmuno.com/catalog/products/705-165-147
Cy3-conjugated donkey anti-rabbit IgG pAbs (711-165-152), https://www.jacksonimmuno.com/catalog/products/711-165-152
FITC-conjugated donkey anti-goat IgG pAbs (705-095-147), https://www.jacksonimmuno.com/catalog/products/705-095-147
FITC-conjugated donkey anti-rabbit IgG pAbs (711-095-152), https://www.jacksonimmuno.com/catalog/products/711-095-152
FITC-conjugated donkey anti-rat IgG pAbs (712-095-153) (Jackson Immunoresearch Laboratories, West Grove, PA). https://www.jacksonimmuno.com/catalog/products/712-095-153
rabbit anti-ERK mAb (1:1000, #4695), Cell Signaling, Danvers, MA.
https://www.cellsignal.com/products/primary-antibodies/p44-42-mapk-erk1-2-137f5-rabbit-mab/4695?Ntk=Products&Ntt=4695&site-search-type=Products
rabbit anti-phosphorylated (p)-ERK mAb (1:2000, #4370), Cell Signaling, Danvers, MA.
https://www.cellsignal.com/products/primary-antibodies/phospho-p44-42-mapk-erk1-2-thr202-tyr204-d13-14-4e-xp-rabbit-mab/4370?Ntk=Products&Ntt=4370
rabbit anti-p38 pAbs (1:1000, #9212), Cell Signaling, Danvers, MA.
https://www.cellsignal.com/products/primary-antibodies/p38-mapk-antibody/9212?Ntk=Products&Ntt=9212
rabbit anti-p-p38 mAb (1:1000, #4511), Cell Signaling, Danvers, MA.
https://www.cellsignal.com/products/primary-antibodies/phospho-p38-mapk-thr180-tyr182-d3f9-xp-rabbit-mab/4511?Ntk=Products&Ntt=4511
rabbit anti-JNK pAbs (1:1000, #9252), Cell Signaling, Danvers, MA.
https://www.cellsignal.com/products/primary-antibodies/sapk-jnk-antibody/9252?Ntk=Products&Ntt=9252
rabbit anti-p-JNK pAbs (1:1000, #9251), Cell Signaling, Danvers, MA.
https://www.cellsignal.com/products/primary-antibodies/phospho-sapk-jnk-thr183-tyr185-antibody/9251?Ntk=Products&Ntt=9251
rabbit anti-GAPDH mAb (1:1000, #5174) Cell Signaling, Danvers, MA.
https://www.cellsignal.com/products/primary-antibodies/gapdh-d16h11-xp-rabbit-mab/5174?Ntk=Products&Ntt=5174
CD11b (1:100, clone M1/70, 20-0112, Tonbo Biosciences, San Diego, CA), https://tonbobio.com/products/apc-anti-human-mouse-cd11b-m1-70
Ly-6G (1:100, clone RB6-8C5, 35-5931, Tonbo Biosciences), https://tonbobio.com/products/fitc-anti-mouse-ly-6g-gr-1-rb6-8c5
F4/80 (1:80, clone BM8.1, 50-4801, Tonbo Biosciences), https://tonbobio.com/products/pe-anti-mouse-f4-80-antigen-bm8-1
B220 (1:50, clone RA3-6B2, 103205, BioLegend, San Diego, CA), https://tonbobio.com/products/fitc-anti-human-mouse-cd45r-b220-ra3-6b2
CD3 (1:40, clone 17A2, 75-0032, Tonbo Biosciences), https://tonbobio.com/products/violetfluor-450-anti-mouse-cd3-17a2
CD4 (1:80, RM4-5, 50-0042, Tonbo Biosciences), https://tonbobio.com/products/pe-anti-mouse-cd4-rm4-5
CD8 (1:80, clone 53-6.7, 20-0081, Tonbo Biosciences), https://tonbobio.com/products/apc-anti-mouse-cd8a-53-6-7
CD45 (1:80, clone 30-F11, 103131, BioLegend), https://www.biolegend.com/en-us/products/percp-cyanine5-5-anti-mouse-cd45-antibody-4264

Eukaryotic cell lines

Policy information about cell lines

Cell line source(s)	Human THP-1 cells were obtained from American Type Culture Collection (Rockville, MD).
Authentication	The cell line is commercial and no authentication has been conducted after purchase.
Mycoplasma contamination	The cell line tested negative for mycoplasma contamination.
Commonly misidentified lines (See ICLAC register)	No misidentified cell lines were used in this study.

Animals and other organisms

Policy information about studies involving animals; ARRIVE guidelines recommended for reporting animal research

Laboratory animals	Specific pathogen-free 8-week-old male C57BL/6 mice were obtained from Japan SLC and designated as wild-type (WT) mice in this study. Homozygous Ccl3-/- mice were obtained from Jackson Laboratories (Bar Harbor, ME). Ccr1-/- mice were a generous gift from Drs. P.M. Murphy and J.L. Gao (National Institute of Allergy and Infectious Diseases, NIH, Bethesda, MD). Ccr5-/- mice were generated as previously described. All these mice were backcrossed to C57BL/6 mice for 10 generations. All mice were kept under the specific pathogen-free conditions at the Animal Research Center of Wakayama Medical University (temperature, 23 ± 1°C; humidity, 50 ± 10%; 12 h light/12 h dark cycle). Age- and sexmatched mice were used for the experiments.
Wild animals	No wild animals were used in this study.
Field-collected samples	No field-collected samples used in this study.
Ethics oversight	All experimental protocols were approved by the Animal Research and Ethics Committee of Wakayama Medical University (approval No. 885). All animal experiments in this study were performed in compliance with the Guidelines for the Care and Use of Laboratory Animals on Wakayama Medical University and with the relevant guidelines and regulations.

Note that full information on the approval of the study protocol must also be provided in the manuscript.

Human research participants

Policy information about studies involving human research participants

Population characteristics	Patients were not discriminated according to age, gender, genotypic information or past/current diagnosis.
Recruitment	Informed consent was obtained for each subject. Samples of unruptured human aneurysmal (>50mm) aortic wall were obtained during open aortic repair procedures or autopsy cases. Aortic wall with no pathological alterations was obtained from autopsy cases.
Ethics oversight	The study using human samples was approved by the Human Ethics Review Committee of Wakayama Medical University (Approval No 2253). All experiments were carried out in compliance with the declaration of Helsinki, the guidelines for ethical principles for medical research involving Human Subjects, the ethical guidelines of Wakayama Medical University, and with the relevant guidelines and regulations.

Note that full information on the approval of the study protocol must also be provided in the manuscript.

Flow Cytometry

Plots

Confirm that:

☒ The axis labels state the marker and fluorochrome used (e.g. CD4-FITC).

☒ The axis scales are clearly visible. Include numbers along axes only for bottom left plot of group (a 'group' is an analysis of identical markers).

☒ All plots are contour plots with outliers or pseudocolor plots.

☒ A numerical value for number of cells or percentage (with statistics) is provided.

Methodology

Sample preparation	Peripheral blood was collected from WT, Ccl3-/-, and Ccr5-/- mice in EDTA tubes to prevent coagulation. Whole peripheral blood<br>(200 µl) samples were stained for flow cytometry.
Instrument	CytoFlex S

Software	FlowJo
Cell population abundance	No sorting was performed.
Gating strategy	Gating strategy is provided in the Supplementary Information.

☒ Tick this box to confirm that a figure exemplifying the gating strategy is provided in the Supplementary Information.
